# An Exploratory Study on the Influence of Frying on Chemical Constituent Transformation and Antioxidant Activity in Ziziphi Spinosae Semen: A Multimodal Analytical Strategy Based on UPLC–Q–TOF–MS and GC–IMS

**DOI:** 10.3390/foods14234145

**Published:** 2025-12-03

**Authors:** Xinyi Ouyang, Xiaonuo Shi, Chang Zhou, Mengyuan Li, Rujia Huang, Huiping Liu, Dan Huang, Guomin Zhang

**Affiliations:** 1 The Key Laboratory of Hunan Province for Integrated Traditional Chinese and Western Medicine on Prevention and Treatment of Cardio-Cerebral Diseases, College of Integrated Traditional Chinese and Western Medicine, Hunan University of Chinese Medicine, Changsha 410208, China; oyxy@stu.hnucm.edu.cn (X.O.); sxn.1106@stu.hnucm.edu.cn (X.S.); zhouchang@stu.hnucm.edu.cn (C.Z.);; 2 Hunan Key Laboratory of Traditional Chinese Medicine Prescription and Syndromes Translational Medicine, Hunan University of Chinese Medicine, Changsha 410208, China; limengyuan@stu.hnucm.edu.cn (M.L.); 003433@hnucm.edu.cn (H.L.); 3 State Key Laboratory of Chinese Medicine Powder and Medicine Innovation in Hunan (Incubation), Academy of Chinese Medical Sciences (Science and Technology Innovation Center), Hunan Province Sino-US International Joint Research Center for Therapeutic Drugs of Senile Degenerative Diseases, Hunan University of Chinese Medicine, Changsha 410208, China

**Keywords:** Ziziphi Spinosae semen, UPLC-Q-TOF-MS, GC-IMS, volatile components, antioxidant activity, medicinal food

## Abstract

Ziziphi Spinosae semen (ZSS) is renowned for its rich nutritional composition and is traditionally consumed in China, Japan, and Korea, where it is widely incorporated into both medicinal diets and daily cuisine. To address the lack of systematic research comparing raw and fried ZSS, this study aimed to elucidate the compositional and functional changes induced by the frying process. This study systematically compared the chemical profiles and antioxidant activities of ZSS and fried ZSS using ultra-performance liquid chromatography–quadrupole–time-of-flight mass spectrometry (UPLC–Q–TOF–MS) and gas chromatography–ion mobility spectrometry (GC–IMS). A total of 92 non-volatile compounds and 43 volatile organic compounds (VOCs) were identified. Frying significantly promoted the formation of polar compounds such as flavonoids and saponins and increased the content of aldehydes and alcohols, thereby generating aromas characteristic of Maillard reactions and lipid oxidation. Principal component analysis (PCA) and orthogonal partial least squares discriminant analysis (OPLS-DA) clearly distinguished the two groups in terms of their chemical composition and flavor characteristics. In addition, 2,2-diphenyl-1-picrylhydrazyl (DPPH) and 2,2′-azino-bis (3-ethylbenzothiazoline-6-sulfonic acid) (ABTS) assays demonstrated that the antioxidant capacity of fried ZSS was significantly higher than that of the raw sample (*p* < 0.05). These results indicate that the frying process reshapes the chemical properties and bioactivity of ZSS via multiple pathways, including glycoside hydrolysis, lipid oxidation, and Maillard reactions. Overall, this study establishes a scientific foundation for the development of functional foods derived from ZSS.

## 1. Introduction

Ziziphi Spinosae semen (ZSS) refers to the mature seeds of *Ziziphus jujuba* Mill. var. *spinosa* (Bunge) Hu ex H. F. Chou [1]. It has a long-standing history of consumption in several East Asian countries, including China, Japan, and Korea, where it has been extensively utilized for over two millennia in Traditional Chinese Medicine (TCM) dietary therapy and daily cuisine, underscoring its historical role as a functional food [2,3,4]. Common foods incorporating ZSS include ZSS tea, ZSS pastries, and ZSS candies. Studies have demonstrated that ZSS exhibits therapeutic properties such as antioxidant, antidepressant, and sleep-improving effects [5,6,7,8]. ZSS is favored by consumers and is characterized by a complex chemical composition rich in saponins, flavonoids, alkaloids, polysaccharides, and volatile oils [9]. It is generally classified into two forms: crude (unprocessed) and stir-fried (processed). Recent studies have suggested that heat treatment may induce chemical transformations that could modify the bioactive constituents and pharmacological properties of ZSS. However, the specific patterns in these chemical changes and their relationship to pharmacological activity have not yet been fully elucidated [5,10].

Volatile compounds in plant-derived food materials serve as critical indicators in assessing product quality and evaluating processing methodologies [11,12]. Previous studies have demonstrated that heat treatment can induce both the Maillard reaction and lipid oxidation in these materials, thereby significantly altering the composition and profile of their volatile components [13,14]. ZSS, being rich in volatile constituents, undergoes changes in both variety and concentration during the stir-frying process; these changes are primarily driven by the Maillard reaction and lipid oxidation, ultimately influencing its flavor profile and functional properties [7]. However, the chemical transformation patterns induced by stir-frying have not been systematically elucidated, and the relationship between these transformations and changes in antioxidant activity remains unclear.

Common analytical techniques for volatile compounds, including headspace gas chromatography–mass spectrometry (HS–GC–MS) and solid-phase microextraction gas chromatography–mass spectrometry (SPME–GC–MS), are widely employed for the effective separation and characterization of key aromatic compounds. However, these methods require rigorous sample preparation procedures [15,16]. Gas chromatography–ion mobility spectrometry (GC–IMS), which requires minimal sample preparation, facilitates the high-throughput characterization of volatile compounds in complex matrices through two-dimensional separation based on both chromatographic retention time and ion mobility drift time. When combined with multivariate statistical analysis, this technique enables the precise identification of characteristic differences among samples [17,18]. Furthermore, ultra-performance liquid chromatography–quadrupole time-of-flight mass spectrometry (UPLC–Q–TOF–MS), distinguished by its high resolution and sensitivity, facilitates the systematic elucidation of compositional variations in non-volatile metabolites, thereby providing robust support for research on the processing mechanisms of plant-derived foods [19,20]. Integrating GC–IMS into UPLC–Q–TOF–MS enables the dual-dimensional analysis of both volatile and non-volatile components in ZSS across various processing methods. This comprehensive approach systematically characterizes the differences in chemical composition and elucidates substance transformation patterns under diverse processing conditions.

Many functional foods rich in volatile compounds exhibit significant antioxidant activity. These bioactive constituents promote health and reduce the risk of various age-related diseases. Antioxidants neutralize reactive oxygen species, thereby mitigating oxidative damage to cellular membranes, proteins, and DNA, preserving both the structural and functional integrity of cells [21]. Consequently, antioxidant activity has emerged as a key indicator in evaluating the functional properties of ZSS and its processed products, with free radical scavenging capacity serving as a direct measure of efficacy within in vitro antioxidant defense systems [22]. Commonly employed assays, such as the DPPH (2,2-diphenyl-1-picrylhydrazyl) and ABTS (2,2′-azino-bis (3-ethylbenzothiazoline-6-sulfonic acid)) radical scavenging tests, quantify activity by measuring reductions in radical absorbance and are known for their simplicity, high sensitivity, and reproducibility [23]. Moreover, existing studies indicate that ZSS oil demonstrates considerable free radical scavenging capacity, and thermal processing may further modulate its antioxidant potential [24].

Stir-frying is a widely used traditional processing method for Ziziphi Spinosae Semen (ZSS) and is considered to influence its chemical composition and overall quality attributes. Although previous studies have reported changes in certain constituents following processing, the overall impact of stir-frying on both volatile and non-volatile components of ZSS has not been systematically characterized, and its relationship with antioxidant activity remains insufficiently understood. To address this gap, the present study applied an integrated analytical strategy combining GC–IMS and UPLC–Q–TOF–MS, together with in vitro antioxidant assays, to comprehensively compare the chemical profiles of raw and stir-fried ZSS. This approach aims to clarify the chemical alterations associated with stir-frying and to provide insight into how these changes may relate to differences in antioxidant capacity.

## 2. Materials and Methods

### 2.1. Materials

ZSS samples were collected from Hebei, China, and authenticated by Professor Zhi Wang at Hunan University of Chinese Medicine. A voucher specimen (HNATCM2025-ZSS) has been deposited in the herbarium of Hunan University of Chinese Medicine.

### 2.2. Preparation of Stir-Fried ZSS

A total of 500 g of ZSS was placed in a stainless-steel stir-frying vessel and processed under standardized dry-frying conditions. The seeds were heated without the addition of oil, using a low flame that produced a surface temperature of approximately 110–120 °C. The material was manually stirred at a consistent rate (approximately 60–80 strokes per minute) to ensure uniform heat exposure and to avoid localized charring. The stir-frying process was maintained for 8–10 min, during which the seeds gradually expanded, developed a slightly deeper surface color, and released a characteristic aroma. When these processing indicators were observed, the material was removed from heat and allowed to cool naturally to room temperature. The untreated seeds were designated as ZSS-01, and the stir-fried product was designated as ZSS-02.

### 2.3. Preparation of Ziziphi Spinosae Semen Oil

A total of 300 g of ZSS was processed using an oil press machine (Bestday Model ZYJ-9029, Jiangmen, Guangdong, China) at pressures reaching 1600 kN while the temperature was carefully maintained between 40 and 60 °C. The oil extracted was subsequently stored in amber glass vials. All oils were kept at −20 °C until the volatile compounds were analyzed using GC–IMS.

### 2.4. Non-Targeted Metabolomics Analysis Based on UPLC-Q-TOF-MS

Only one independent raw (ZSS-01) and one stir-fried (ZSS-02) sample were available for non-targeted metabolomics. Accordingly, a single analytical injection was performed for each sample. The metabolomic results therefore reflect qualitative rather than statistically replicated quantitative comparisons.

#### 2.4.1. Preparation of Sample Solutions

A 1.0 g sample of either ZSS or its stir-fried powder was precisely weighed and placed into individual containers. Then, 10 mL of 80% methanol was added to each sample. The mixtures were subsequently subjected to ultrasonic extraction at 300 W and 40 kHz for 30 min. After cooling, each mixture was thoroughly agitated by shaking. A 2 mL portion of each extract was centrifuged at 12,000 r/min for 5 min, with the resulting supernatant collected as the test solution.

#### 2.4.2. Preparation of Reference Standard Solutions

Standard reference compounds—including coclaurine, magnoflorine, vicenin-2, spinosin, and betulinic acid—were precisely measured and dissolved in methanol to create a combined reference solution. The mixture was then centrifuged at 12,000 rpm for 5 min, and the supernatant was collected for further analysis. These standards were later used to confirm compound identities by comparing their chromatographic retention times and fragment ion patterns.

#### 2.4.3. Chromatographic Conditions

Chromatographic separation was performed using an Agilent ZORBAX SB-Aq column (2.1 × 100 mm, 1.8 µm) (Agilent Technologies,  Santa Clara, CA, USA) maintained at 35 °C. The mobile phase consisted of (A) 0.1% formic acid in water and (B) acetonitrile. The gradient elution program was as follows: 0–8 min, 3%–16% B; 8–20 min, 16%–28% B; 20–24 min, 28%–56% B; 24–32 min, 56%–75% B; 32–40 min, 75%–95% B; 40–43 min, 95% B; 43–43.1 min, 95%–3% B; 43.1–46 min, maintain 3% B. The flow rate was set at 0.3 mL/min with an injection volume of 2 µL. Detection was conducted at 270 nm, and full-wavelength UV spectra were acquired from 190 to 400 nm.

#### 2.4.4. Mass Spectrometric Conditions

The analysis was performed using an AB Sciex Triple TOF^®^ 4600 high-resolution mass spectrometer fitted with an electrospray ionization (ESI) source, functioning in both positive- and negative-ion modes. The ion source was kept at 500 °C, with spray voltages of +5000 V for positive mode and −4500 V for negative mode. The nebulizing and auxiliary gases were both maintained at 50 psi, while the curtain gas was regulated at 35 psi. The full mass scan covered an *m*/*z* range of 50–1700, and the MS/MS acquisition spanned *m*/*z* 50–1250. Additionally, a collision energy of ±40 eV with an energy spread of 20 eV was applied to enable stepped collision-induced dissociation.

### 2.5. Analysis of Volatile Components Using GC-IMS

Volatile organic compounds (VOCs) in ZSS-01 and ZSS-02 oils were analyzed using GC–IMS. The analyses were performed using a FlavourSpec^®^ GC–IMS instrument (G.A.S., Dortmund, Germany) equipped with a CTC-PAL 3 static headspace autosampler, and data were processed using VOCal software (version 0.4.10).

A 1.0 mL aliquot of ZSS oil was precisely dispensed into a 20 mL headspace vial, which was then sealed and loaded into the headspace autosampler. For each condition, one independent oil sample was prepared, and each sample was analyzed with three technical replicates following the standard GC–IMS workflow. The vial was incubated at 60 °C for 15 min with agitation at 500 rpm. A 200 µL sample was injected in splitless mode, with the injection needle maintained at 110 °C. Gas chromatography separation was achieved using an MXT-WAX capillary column (15 m × 0.53 mm × 1.0 μm, Restek Inc., Bellefonte, PA, USA) held at a constant 60 °C. High-purity nitrogen (≥99.999%) served as the carrier gas with a programmed flow rate: beginning at 2.0 mL/min for 2 min, linearly increasing to 10.0 mL/min over the next 8 min, then ramping up to 100.0 mL/min over an additional 10 min, and finally sustaining 100.0 mL/min for 30 min, resulting in a total analysis time of 50 min.

Ion mobility spectrometry was conducted using a tritium (^3^H) ionization source. The drift tube, measuring 53 mm in length and maintained at 45 °C, operated under an electric field of 500 V/cm. High-purity nitrogen (99.999%) was utilized as the drift gas at a flow rate of 75.0 mL/min, with detection performed in positive-ion mode.

Compound identification by GC–IMS: Tentative identification of volatile compounds was performed using VOCal software (version 0.4.10, G.A.S., Dortmund, Germany). A homologous series of ketone standards (2-butanone to 2-nonanone, C_4_–C_9_) was analyzed to generate the calibration curve for converting gas chromatographic retention times into retention indices (RI). For each detected VOC feature, the experimentally derived RI was calculated and subsequently matched against both the built-in GC retention-index database (NIST RI database, 2020 edition, integrated in VOCal) and the proprietary IMS drift-time library integrated within VOCal. Compound assignments were accepted only when the RI and drift-time values simultaneously met the manufacturer-defined similarity thresholds. As no authentic chemical standards were individually analyzed to verify peak identities, all VOC annotations in this study should be regarded as putative (MSI level 2–3), based solely on combined RI and drift-time library matching rather than definitive structural confirmation.

### 2.6. Evaluation of In Vitro Antioxidant Activity of Ziziphi Spinosae Semen Oil

The in vitro antioxidant activity of Ziziphi Spinosae semen oil (ZSSO) was assessed using DPPH and ABTS radical scavenging assays. The DPPH radical scavenging activity was determined utilizing a DPPH Radical Scavenging Capacity Assay Kit (Biosharp Life Sciences, Hefei, China), whereas the ABTS activity was measured with an ABTS Radical Scavenging Capacity Assay Kit (ADS Biotechnology Co., Ltd., Yancheng, China). ZSSO samples were dissolved in anhydrous ethanol and prepared at various mass concentrations. Absorbance readings were taken at 517 nm and 734 nm according to the respective kit instructions, and the radical scavenging rate (R) was calculated using the following formula:

R = [(1 − (A_0_ − A_1_) ÷ A_1_)] × 100%

Ascorbic acid (Vc) was used as the positive control. The half maximal inhibitory concentration (IC_50_, mg/mL) was determined based on the dose–response relationship between the sample concentration and scavenging rate.

### 2.7. Statistical Analysis

Data collection via UPLC-Q-TOF-MS was conducted using Analyst TF 1.7.1 software (AB Sciex, Framingham, MA, USA), with subsequent data processing performed in PeakView 1.2 (AB Sciex, USA). The resulting mass spectra were matched against Natural Products HR-MS/MS Spectral Library 1.0, and identifications were further corroborated by consulting the literature and examining fragment ion characteristics. For compounds with available reference standards, retention times and specific fragment ions were compared to further confirm identification accuracy and ensure reliability. Prior to conducting chemometric analyses, the UPLC-Q-TOF-MS data were normalized by peak area and subjected to logarithmic transformation. Based on the relative signal intensities, the 30 most abundant compounds that contributed significantly to sample variation were chosen to generate scatter plots and bubble charts, thereby visualizing the distinct distribution patterns and compound class features across the samples.

GC-IMS data were evaluated using pattern recognition and differential analysis techniques, specifically principal component analysis (PCA) and orthogonal partial least squares discriminant analysis (OPLS-DA). PCA was performed with the prcomp function in R, which helped to reveal the variance structure and overall distribution characteristics among the samples. Meanwhile, an OPLS-DA model was built using SIMCA 14.1 (Umetrics, Umeå, Sweden), and key differential compounds were pinpointed by examining variable importance in projection (VIP) values, with a threshold of VIP > 1 indicating significance. Additionally, three-dimensional spectra, two-dimensional differential spectra, and fingerprint maps were produced using VOCal software (version 0.4.10, G.A.S., Dortmund, Germany). Further, volcano plots were created in the R environment with ggplot2, applying screening criteria of |log_2_FC| > 0.3 and *p* < 0.05 to clearly display the up- and downregulation patterns of volatile organic compounds across various processing methods.

The DPPH and ABTS radical scavenging assay results are presented as means ± standard deviation (*n* = 3). Statistical evaluations were conducted using SPSS version 26.0, employing analysis of variance, with the significance threshold set at *p* < 0.05.

All graphical visualizations were produced in the R environment using packages including ggplot2, ropls, and tidyverse.

## 3. Results

### 3.1. Analysis of UPLC-Q-TOF-MS Detection Results

#### 3.1.1. UPLC-Q-TOF-MS Component Identification Results

**Figure 1:**
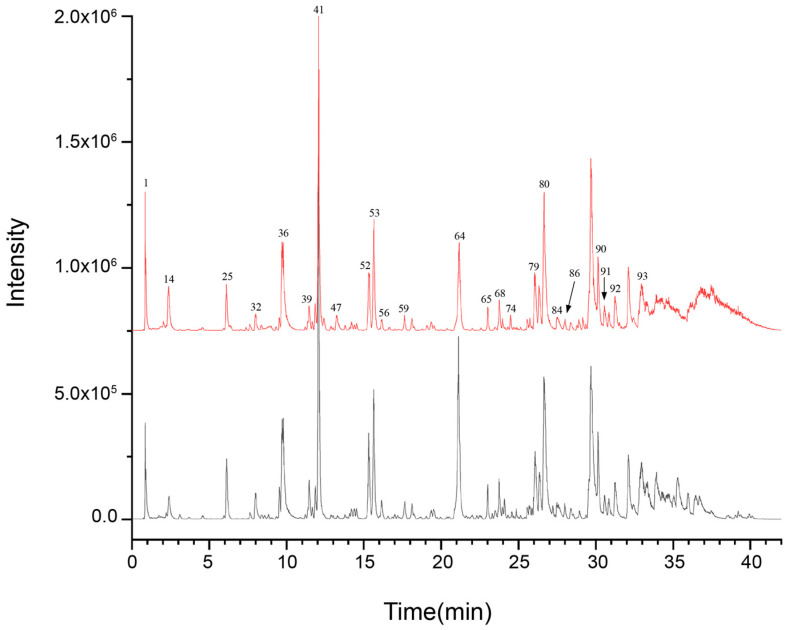
UPLC-Q-TOF-MS base peak chromatogram (BPC) for ZSS-1 and ZSS-2 in negative ionization mode.

**Figure 2:**
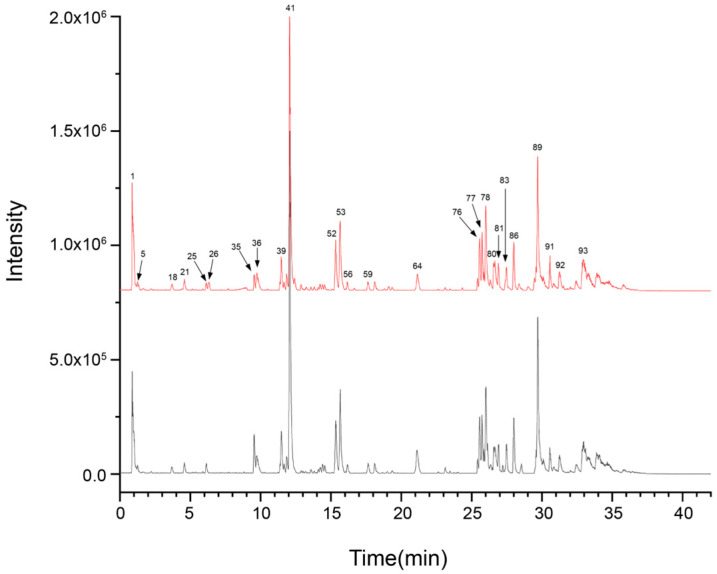
UPLC-Q-TOF-MS base peak chromatogram (BPC) for ZSS-01 and ZSS-02 in negative ionization mode.

In order to clarify the major chemical components present in ZSS oil and to examine how stir-frying influences its chemical makeup, UPLC-Q-TOF-MS was utilized to profile both ZSS and stir-fried ZSS samples. The data were collected using Analyst TF 1.7.1 software, while PeakView 1.2 was used for processing. Initially, the mass spectrometry findings were matched with the Natural Products HR-MS/MS Spectral Library 1.0 database, supplemented by a review of the relevant literature and analysis of fragmentation patterns. For compounds for which reference standards were accessible, identification was further validated by cross-referencing retention times and characteristic fragment ions to ensure precision and reliability. The resulting base peak chromatograms (BPCs) in both positive and negative ionization modes, depicted in Figure 1 and Figure 2, respectively, are well-defined.

A total of 81 unique compounds were identified in the raw ZSS samples, and 92 unique compounds were detected in the stir-fried samples. Importantly, certain metabolites were detected in more than one ion form—such as structural isomers, adduct ions, or in-source fragment ions—which are listed as separate analytical entries in Table 1. For the purpose of compound enumeration, these ion-related entries were consolidated and counted as single metabolites. Consequently, the total number of table entries (n = 93) does not equate directly to the number of unique compounds reported. All ion forms and corresponding annotations are presented in detail in Table 1.

A qualitative comparison of the 81 metabolites detected in raw ZSS and the 92 metabolites observed in stir-fried ZSS suggests substantial overlap while also indicating potential processing-related differences. Based on the current dataset, approximately 71 compounds appear to be shared between the two samples, implying that most constituents remained detectable after stir-frying. In contrast, around 10 metabolites were found only in raw ZSS, which may reflect thermal degradation, volatilization, or diminished detectability following processing. Conversely, approximately 21 metabolites were detected exclusively in the stir-fried sample, possibly due to heat-induced formation, enhanced extractability, or matrix-associated modifications that increase ionization efficiency. Among the shared metabolites, several exhibited notable shifts in relative peak intensity (including vicenin-2, spinosin, coclaurine, magnoflorine, catechin, and multiple fatty-acid–derived compounds). These trends may be consistent with heat-associated processes such as glycosidic bond cleavage, oxidation, or structural rearrangement. Although the absence of technical replicates limits the strength of quantitative interpretation, the overall pattern suggests that the larger number of detected metabolites in ZSS-02 likely reflects processing-related compositional changes, rather than analytical variability alone. These changes may encompass the partial loss of thermolabile compounds, the emergence of new or newly detectable constituents, and modifications in the abundance of metabolites shared between raw and stir-fried ZSS.

#### 3.1.2. Qualitative Comparison of Component Distribution and Chemical Category Differences

**Figure 3:**
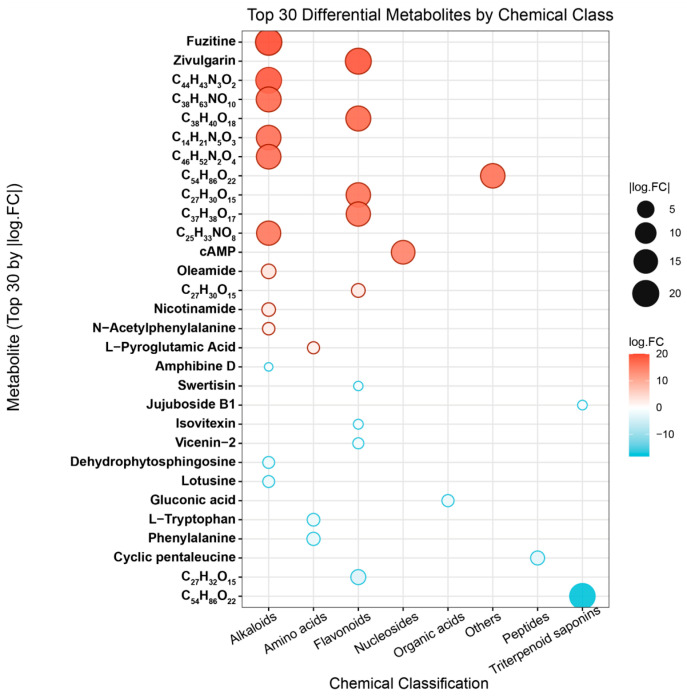
Top 30 differential metabolites classified by chemical classes.

To further investigate the differences in chemical composition between ZSS-01 and ZSS-02, a non-targeted metabolomic analysis was performed using UPLC-Q-TOF-MS. Figure 3 shows the 30 most representative metabolites, characterized by strong detection signals, that were selected for visualization. The analysis showed that the metabolite profiles of the two ZSS oil groups differed markedly both in type and abundance. Specifically, the ZSS-02 exhibited notably higher signal intensities for several flavonoids (e.g., fuzitine and apigenin glucosides) and saponins, whereas the cold-pressed sample (ZSS-01) contained relatively greater amounts of certain organic acids and amino acid derivatives (e.g., l-pyroglutamic acid and phenylalanine). These disparities are likely due to processes such as glycosidic bond cleavage, the deconjugation of phenolic acids, and mild lipid oxidation during stir-frying, which facilitate the release and structural rearrangement of bound compounds.

**Figure 4:**
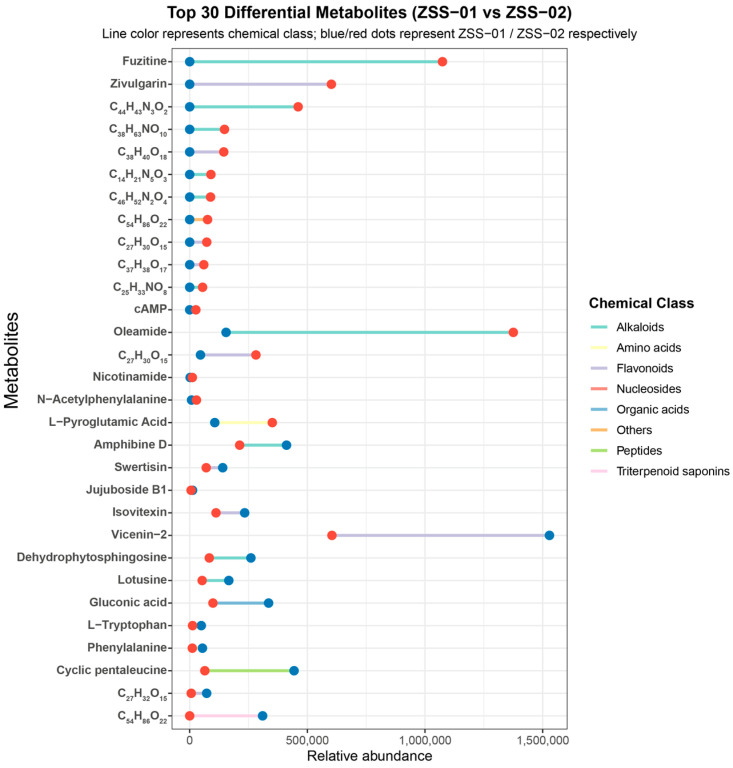
Top 30 differential metabolites (ZSS-01 vs. ZSS-02) showing relative abundance comparison.

Further categorization based on chemical class (Figure 4) revealed that flavonoids, saponins, organic acids, and amino acids are the predominant metabolite groups. Notably, stir-frying enhanced the relative levels of flavonoids and triterpenoid saponins, while the cold-pressed technique preserved higher concentrations of organic acids and amino acids. These trends suggest that the thermal conditions during stir-frying promote the deglycosylation of flavonoid glycosides and the rearrangement of triterpenoid skeletons, ultimately altering the chemical composition and proportion of bioactive constituents in ZSS oil. These chemical changes likely contribute to the enhanced antioxidant activity observed subsequently.

### 3.2. Analysis of GC-IMS Detection Results

#### 3.2.1. Analysis of Volatile Component Differences in Ziziphi Spinosae Semen Oil with Different Processing Methods

**Figure 5:**
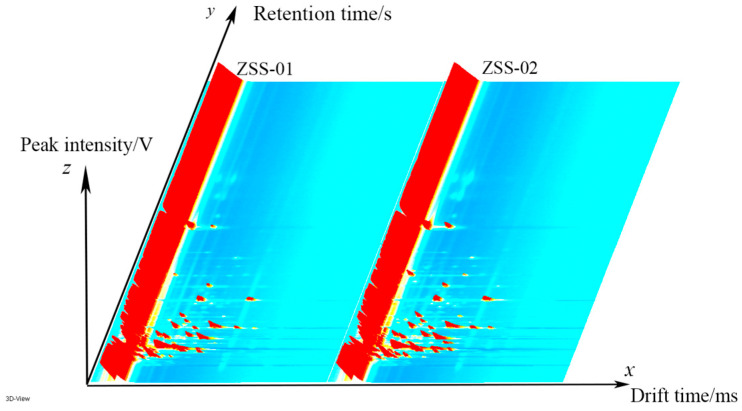
Three-dimensional GC–IMS spectra of volatile organic compounds (VOCs) in ZSS-01 and ZSS-02 oils. The color scale represents signal intensity, with blue indicating low intensity and red indicating high intensity.

Additionally, GC–IMS technology was utilized to assess the volatile oils extracted from both ZSS-01 and ZSS-02. By employing the Reporter plug-in in VOCal data processing software, we generated three-dimensional, two-dimensional, and differential spectra that illustrate alterations in volatile components. Figure 5 illustrates a three-dimensional spectrum where the X-axis represents drift time, the Y-axis corresponds to gas chromatography retention time, and the Z-axis reflects signal peak intensity. This visualization offers a clear depiction of the volatile distribution, with red peaks indicating regions with high VOC concentrations and blue-green areas signifying lower intensities.

**Figure 6:**
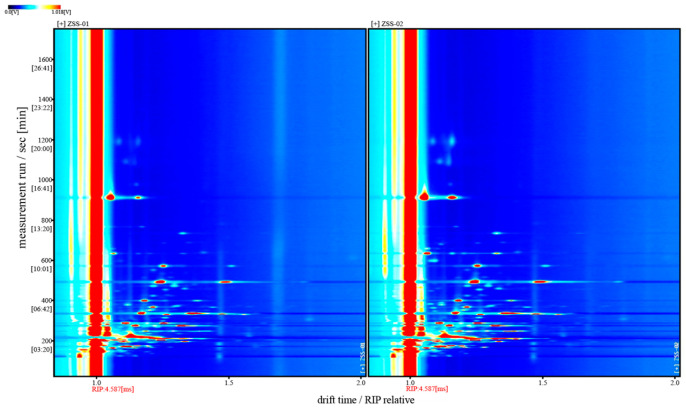
Two-dimensional GC–IMS spectra of volatile organic compounds (VOCs) in ZSS-01 and ZSS-02 oils. Note: Vertical markers indicate the drift-time start positions for ZSS-01 and ZSS-02, respectively.

For a clearer comparative view, Figure 6 displays the two-dimensional top-view GC–IMS spectra of ZSS-01 and ZSS-02, with the horizontal axis representing the drift time and the vertical axis showing the retention time. The color intensity reflects the signal strength; red indicates high concentrations, and blue represents low ones. The reactive ion peak (RIP) appears near 1.0 on the X-axis, and the surrounding-colored spots represent signals from specific volatile compounds. The marked differences in the number and distribution of peaks between the samples underscore substantial variations in their VOC profiles.

**Figure 7:**
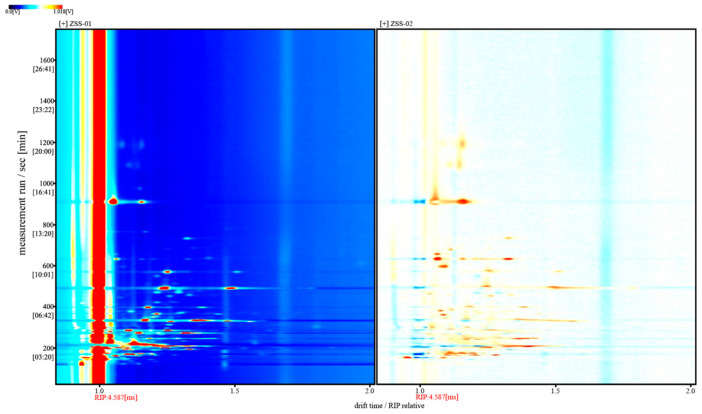
Differential GC–IMS spectra of volatile components in ZSS-01 and ZSS-02 oils.

Figure 7 presents the differential GC–IMS spectrum obtained by subtracting the signal intensities of ZSS-01 from those of ZSS-02. In this visualization, white regions indicate negligible differences between the two samples, whereas red and blue represent positive and negative deviations, respectively. The dominance of red signals arises from the consistently higher overall intensities observed in ZSS-02 across broad drift-time and retention-time ranges. Accordingly, the asymmetric color distribution reflects authentic increases in volatile constituents in ZSS-02 rather than inconsistencies in the visual encoding. These results further substantiate that the stir-frying process enhances the formation or release of specific volatile compounds.

**Figure 8:**
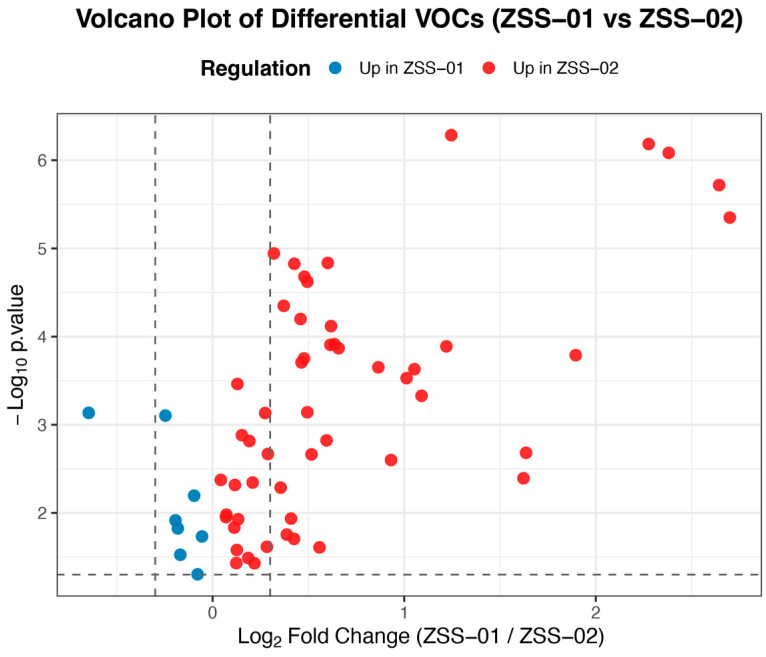
Volcano plot of differential volatile organic compounds (VOCs) between ZSS-01 and ZSS-02 oils.

A differential analysis of VOCs based on GC–IMS data is presented as a volcano plot (Figure 8). In this plot, the horizontal axis shows the log_2_ fold change in relative abundance between the groups, and the vertical axis indicates the significance level (−log_10_ *p*-value). Red dots mark compounds with higher abundance in the stir-fried ZSS oil (ZSS-02), while blue dots correspond to those more prevalent in the cold-pressed oil (ZSS-01). The overall increase in the abundance of most volatile compounds after stir-frying suggests that thermal treatment may induce lipid oxidation and Maillard reactions, thereby reshaping the aroma profile of the oil.

#### 3.2.2. Analysis of GC–IMS Volatile Organic Components in Ziziphi Spinosae Semen Oil with Different Processing Methods

As summarized in Table 2, by integrating GC retention indices with IMS drift time databases, a total of 54 signal entries were detected in the GC–IMS topographic plots. Several compounds appeared as monomer/dimer signal pairs (annotated as “–M” and “–D”), which represent the same chemical substance. After merging these isomeric signal pairs, a total of 43 unique volatile components were obtained. These 43 compounds mainly encompass alcohols (8), aldehydes (8), esters (7), ketones (6), terpenes (4), carboxylic acids (3), as well as pyrazines, furans, and sulfur-containing compounds. Clear differences were observed between raw (ZSS-01) and stir-fried (ZSS-02) oils. Several alcohols such as 1-propanol, 1-pentanol, and 3-methylbutan-1-ol exhibited noticeable changes after stir-frying. Fatty-acid-oxidation-related aldehydes such as 1-hexanal, heptaldehyde, butanal, and propanal generally decreased after stir-frying, likely due to thermal degradation or volatilization. Several ester compounds, including methyl acetate and ethyl 2-methylpropionate, increased in the stir-fried sample, consistent with heat-enhanced esterification reactions. Terpenoid compounds such as α-terpinene and γ-terpinene were more abundant after stir-frying, indicating enhanced release of aroma-active terpenes. Furan derivatives and pyrazines—particularly 2-methylpyrazine and 2,5-dimethylpyrazine—showed strong increases in ZSS-02, reflecting typical Maillard-reaction products generated during heating. Taken together, GC–IMS profiling indicates that stir-frying substantially reshapes the volatile landscape of ZSSSO by reducing lipid-oxidation aldehydes and increasing Maillard-derived pyrazines and aroma-releasing terpenes, which may jointly contribute to the enhanced flavor characteristics of the processed product.

#### 3.2.3. Multivariate Statistical Analysis

**Figure 9:**
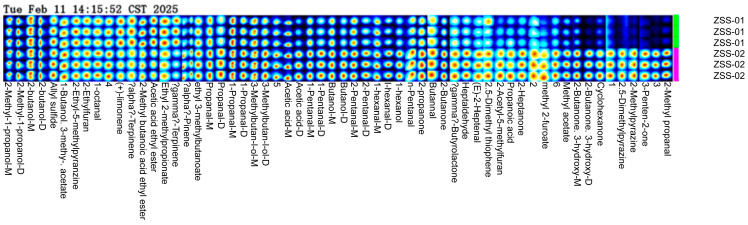
GC–IMS fingerprint profiles of volatile organic compounds (VOCs) found via screening of ZSS-01 and ZSS-02 oils. Color intensity corresponds to signal intensity, with warmer colors indicating higher compound abundance.

To further explore how processing methods affect the volatile components in ZSS oil, a fingerprint analysis was conducted using GC–IMS results from two sample groups (see Figure 9). This fingerprint map visually represents the peak intensities and distribution of all detected volatile compounds. Each row denotes the complete VOC signal profile of an individual sample, while each column reflects the variation in the same VOC’s peak intensity across different samples. Higher relative concentrations are shown using brighter colors. There are noticeable differences in both the type and abundance of volatile compounds between ZSS-01 and ZSS-02. Specifically, the ZSS-01 sample displayed stronger signals for terpenes and esters—such as (+)-limonene, α-terpinene, γ-terpinene, ethyl 2-methylpropionate, and acetic acid ethyl ester—which contribute to a fresh, fruity, and light aroma. In contrast, the ZSS-02 sample was richer in aldehydes and alcohols including α-pinene, ethyl 3-methylbutanoate, propanal, 1-propanol, 3-methylbutan-1-ol, 1-pentanol, butanal, n-pentanal, and 1-hexanol, imparting a more pronounced alcoholic and oily flavor. Moreover, oxygen-containing heterocyclic and ketone compounds—such as 2-butanone, 2-heptanone, γ-butyrolactone, 2-acetyl-5-methylfuran, and 2,5-dimethylpyrazine—were detected in ZSS-02, suggesting that the stir-frying process might promote the formation or release of these distinctive flavor compounds.

These observations indicate that stir-frying significantly alters the flavor profile of ZSS oil. Raw ZSS better retains its natural terpenes and esters, which add fresh, aromatic notes, while stir-frying enhances the presence of aldehydes and alcohols, potentially leading to a more intense and robust flavor. The high-resolution fingerprint map thus provides valuable information for quality control and process optimization in ZSS oil.

**Figure 10:**
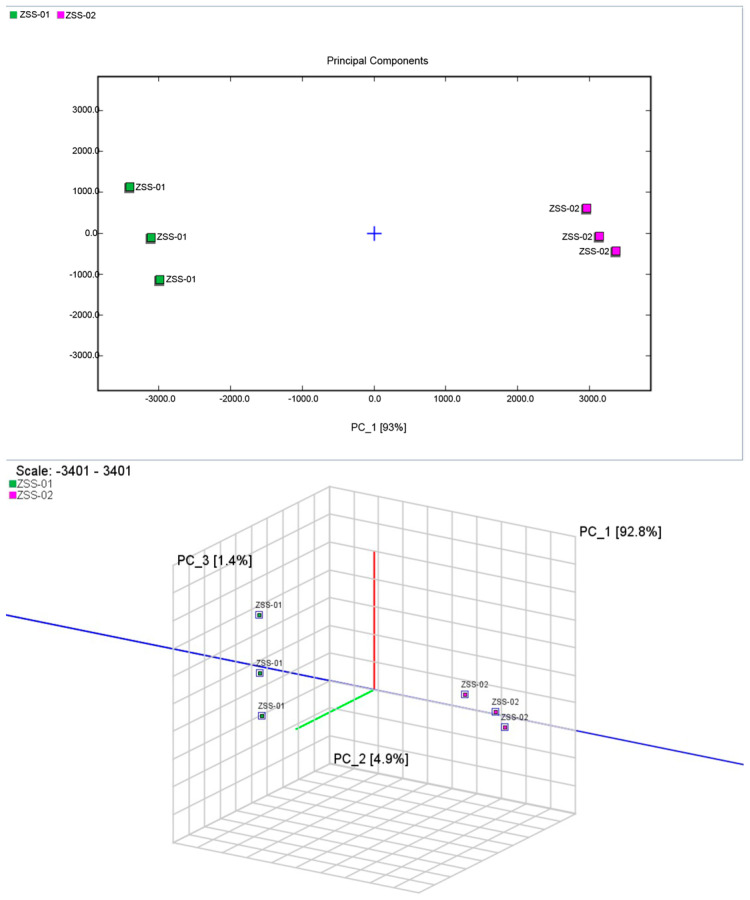
Principal component analysis (PCA) score plot for volatile organic compounds (VOCs) in ZSS-01 and ZSS-02 oils.

Using the peak intensity data from GC–IMS, chemometric techniques were employed for multidimensional statistical analysis to clarify the overall effects of different processing methods on the volatile composition of ZSS oil. Principal component analysis (PCA), a widely used unsupervised method, was applied to reduce the data’s dimensionality by transforming it into several principal components, thereby revealing clustering trends and differences among samples. After normalizing all original signals, these were modeled using the Dynamic PCA module in VOCal software (version 0.4.10, G.A.S., Germany), with the resulting two-dimensional and three-dimensional score plots presented in Figure 10.

In the two-dimensional PCA score plot, PC1 and PC2 contribute 93.0% and 5.0% of the total variance, respectively, meaning that combined, they explain 98.0% of the overall variability among the samples. The three-dimensional PCA model further increased the cumulative contribution, with PC1, PC2, and PC3 accounting for 99.1% of the variance—with PC1 alone contributing 92.8%—indicating its dominant influence. The score plots clearly separate the cold-pressed (ZSS-01) samples from the stir-fried plus cold-pressed (ZSS-02) ones, with each group showing tight clustering and high reproducibility. This clear segregation suggests that the processing methods greatly influence the VOC composition, with the separation along the PC1 axis being particularly significant.

Through combining these PCA findings with the GC–IMS differential spectra and fingerprint maps, it becomes evident that stir-frying markedly modifies the pattern of volatile compounds in ZSS oil, systematically separating the samples within the flavor–chemical space.

**Figure 11:**
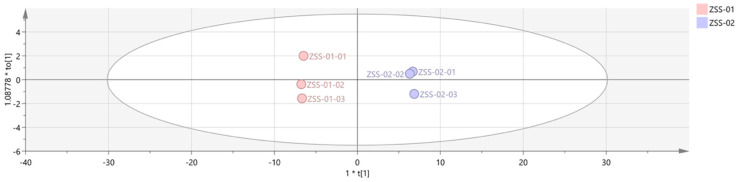
OPLS−DA analysis of VOCs in 2 groups of samples.

Orthogonal partial least squares discriminant analysis (OPLS–DA) was then employed to construct a discriminant model, known for its strong cross-validation performance and enhanced clarity [25]. The model’s goodness-of-fit and explanatory capacity were evaluated using R^2^X and R^2^Y, while Q^2^ measured its predictive power [26]. Values above 0.5 are generally acceptable, with those near 1 indicating an excellent fit and robust prediction capability. An OPLS-DA model was built using SIMCA software with data from both ZSS-01 and ZSS-02 samples. As shown in Figure 11, the model parameters were R^2^X = 0.967, R^2^Y = 0.999, and Q^2^ = 0.998, which demonstrates its high predictive accuracy and excellent fit.

**Figure 12:**
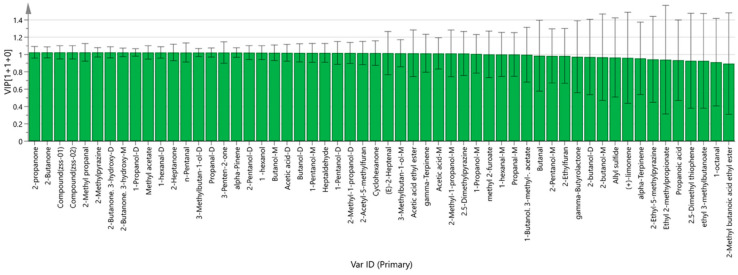
VIP values of the characteristic variables.

Based on the OPLS-DA model, the variable importance in projection (VIP) for each volatile compound was calculated to determine its role in differentiating the groups. Compounds with VIP scores greater than 1 were considered significant. As illustrated in Figure 12, components such as (E)-2-Heptenal, 1-hexanol, 1-hexanal-D, 1-hexanal-M, 1-Pentanol-D, 1-Pentanol-M, 1-Propanol-D, 1-Propanol-M, 2,5-Dimethylpyrazine, 2-Acetyl-5-methylfuran, 2-Butanone, 3-hydroxy-2-Butanone (both D and M forms), 2-Heptanone, 2-Methyl propanal, 2-Methyl-1-propanol (D and M forms), 2-Methylpyrazine, 2-Pentanol-D, 2-propanone, 3-Methylbutan-1-ol (D and M forms), 3-Penten-2-one, acetic acid ethyl ester, acetic acid (D and M forms), alpha-Pinene, Butanol (D and M forms), Cyclohexanone, gamma-Terpinene, Heptaldehyde, methyl 2-furoate, methyl acetate, n-Pentanal, and propanal (D and M forms) may serve as key markers in distinguishing between Ziziphi Spinosae semen oils produced via different processing methods.

**Figure 13:**
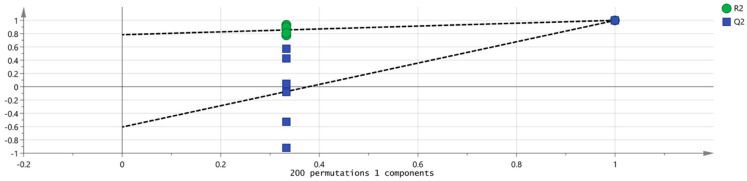
Permutation test results of VOCs in 2 groups of samples.

To assess potential overfitting and confirm the model’s robustness, 200 permutation tests were conducted. As indicated in Figure 13, the R^2^ and Q^2^ values from the permuted data were lower than the original values at x = 1.0, and the Q^2^ regression line intercepted the x-axis with a negative value. This confirms that the model is statistically valid and robust, without signs of overfitting.

### 3.3. Analysis of Antioxidant Activity

This study assessed the DPPH and ABTS radical scavenging activities of two ZSS oil samples, labeled ZSS-01 and ZSS-02, using ascorbic acid (Vc) as a positive control. The results are presented in Table 3.

In the DPPH assay, the IC_50_ values for both ZSS oil samples were in the mg/mL range, markedly exceeding that of Vc (6.83 ± 0.60 μg/mL), which suggests a comparatively lower antioxidant potency. Notably, ZSS-02 showed a significantly lower DPPH IC_50_ value (5.74 ± 0.20 mg/mL) than ZSS-01 (9.27 ± 0.84 mg/mL, *p* < 0.05). This reduction implies that a controlled stir-frying process enhances the oil’s ability to neutralize free radicals. This enhancement may result from the breakdown of glycosidic bonds, the liberation of phenolic acids, and the thermal conversion of antioxidant precursors such as saponins or flavonoids during stir-frying.

Similarly, the ABTS assay mirrored the DPPH results. ZSS-02 exhibited a significantly lower ABTS IC_50_ value (8.12 ± 0.31 mg/mL) compared to ZSS-01 (9.33 ± 0.25 mg/mL, *p* < 0.05), further indicating that stir-frying boosts the ABTS radical scavenging activity of ZSS oil. Meanwhile, ascorbic acid demonstrated a much lower ABTS^+^ IC_50_ value of 2.8 ± 0.10 μg/mL, underscoring the method’s sensitivity and its utility as a reference for antioxidant activity evaluation.

Overall, the findings from both the DPPH and ABTS assays reveal that the stir-fried sample possesses a superior antioxidant capacity relative to ZSS-01. It appears that thermal reactions, such as the Maillard reaction and mild lipid oxidation during stir-frying, promote the formation and concentration of antioxidant compounds—including phenolics, flavonoids, and free fatty acids—thereby enhancing the oil’s free radical scavenging capability. These observations are consistent with previous studies, which have suggested that moderate thermal treatment can enhance the inherent antioxidant properties of plant oils.

In conclusion, the antioxidant efficacy of ZSS oil is closely linked to its processing method. Stir-frying has been shown to significantly augment its free radical scavenging activity, providing valuable insights for optimizing processing techniques and advancing the functional development of ZSS oil.

## 4. Discussion

Processing methods are among the critical factors influencing the chemical composition and quality characteristics of plant-derived foods. As a traditional resource that is both medicinal and edible, ZSS is typically processed by stir-frying. However, systematic investigations into compositional changes and functional alterations in its lipid fraction have been limited. In the present study, UPLC–Q–TOF–MS and GC–IMS techniques were employed to compare the chemical profiles and antioxidant activities of ZSS and stir-fried ZSS. The results demonstrated that stir-frying significantly modified both the chemical and flavor characteristics of ZSS.

UPLC–Q–TOF–MS analysis (Table 1; Figure 1 and Figure 2) revealed a broader non-volatile metabolite spectrum in stir-fried ZSS (ZSS-02) compared with raw ZSS (ZSS-01), with notable enrichment across flavonoids, saponins, triterpenoids, phenolic acids, and amino-acid–derived metabolites. These changes correspond closely to several well-documented thermochemical pathways that occur during heat processing of plant-based matrices. First, thermal cleavage of glycosidic bonds leads to the release of flavonoid aglycones and deglycosylated saponins—reactions widely reported in the processing of Cordyceps, ginseng, Polygonatum, and other medicinal materials [27]. Thermal exposure may additionally activate esterification, oxidation, and de-esterification pathways, promoting the conversion of matrix-bound constituents into more readily extractable or ionizable free forms—such as phenolic acids and flavonoid aglycones—which explains the enhanced peak intensities observed in ZSS-02 (Figure 4) [28]. At the same time, mild oxidative conditions facilitate the esterification, oxidation, and de-esterification of triterpenoid and phenolic precursors, generating oxidized or esterified derivatives with improved chromatographic detectability [16]. Additionally, heat disrupts cell-wall polysaccharides and lipid–phenolic complexes, liberating previously bound metabolites into more extractable or ionizable free forms. Together, these thermally driven pathways account for the coordinated increase in multiple metabolite classes following stir-frying [29]. These observations are consistent with previous studies indicating that moderate thermal treatment enhances the liberation, transformation, and diversification of bioactive constituents in plant-derived materials, thereby contributing to the increased chemical complexity characteristic of stir-fried ZSS.

A total of 43 VOCs were identified using GC–IMS. The three-dimensional topographic plots (Figure 5) and two-dimensional ion mobility spectra (Figure 6) clearly illustrate an increase in both the abundance and distribution of VOCs in ZSS-02 compared with ZSS-01. Moreover, the differential spectrum generated by spectral subtraction (Figure 7) highlights extensive red regions in the stir-fried sample, indicating that numerous VOCs exhibited enhanced signal intensities following thermal processing. A total of 43 VOCs were identified across the two preparation methods (Table 2), and fingerprint matrices (Figure 9) reveal that although most VOCs are shared, their relative abundances differ substantially. Volcano plot analysis (Figure 8) further confirms this trend, with the majority of differential VOCs upregulated in ZSS-02, whereas a small subset—primarily terpenes and esters—were enriched in ZSS-01. The elevated aldehydes in ZSS-02, including hexanal, heptanal, and (E)-2-heptenal, are characteristic lipid oxidation products derived from the thermal degradation of linoleic and oleic acids [30]. These findings align with previous studies demonstrating increased aldehyde formation during the roasting or stir-frying of lipid-containing seeds and animal fats [31]. Additionally, the rise in 1-pentanol, 2-heptanone, cyclohexanone, and 2-methylpyrazine suggests the concurrent contribution of Maillard reactions, these compounds indicate that ZSS undergoes typical thermally induced reactions during stir-frying, resulting in a more complex aromatic profile. Principal component analysis (Figure 10) demonstrates clear separation between ZSS-01 and ZSS-02, supporting that thermal processing induces a systematic shift in VOC composition. Collectively, the GC–IMS results demonstrate that stir-frying promotes lipid oxidation, Maillard reactions, and structural degradation/rearrangement of volatile precursors, thereby enhancing the complexity and intensity of the aroma profile of ZSS oil [32].

As shown in the PCA score plot (Figure 10), raw (ZSS-01) and stir-fried samples (ZSS-02) clustered into two clearly separated groups with minimal intragroup variation, indicating that stir-frying exerts a dominant influence on VOC patterning. This separation was further corroborated by the 3D-PCA model (Figure 10), which demonstrated similarly high cumulative explained variance, reinforcing the robustness of sample discrimination. To more precisely evaluate class separation, OPLS-DA was applied. The OPLS-DA score plot (Figure 11) revealed an even more pronounced intergroup distinction, while the corresponding permutation test (Figure 12) confirmed that the model was not overfitted, given the markedly lower permuted Q^2^ values relative to the original model. The VIP ranking (Figure 12) highlighted (E)-2-heptenal, cyclohexanone, heptanal, and ethyl acetate as the major contributors to between-group variance. These VOCs are established products of lipid oxidation and Maillard-derived reactions, further supporting that thermally driven pathways play a central role in aroma diversification during stir-frying. Taken together, the integration of GC–IMS with chemometric modeling effectively visualized the processing-induced reorganization of VOC networks in ZSS oil. The consistent clustering behavior, stable model validation, and high-impact VOC markers collectively demonstrate that stir-frying generates a more oxidized and thermally enriched aromatic profile. Beyond characterizing compositional shifts, these analyses also illustrate the analytical potential of GC–IMS–chemometric workflows for establishing rapid, non-destructive evaluation models for ZSS quality differentiation under varying processing conditions, consistent with the broader literature on thermal modulation of lipid-derived aroma systems [33].

The results of the DPPH and ABTS assays (Table 3) showed that ZSS-02 exhibited lower IC_50_ values than ZSS-01, indicating a relatively stronger radical-scavenging capacity following stir-frying. Although the differences between the two samples reached statistical significance, their magnitudes observed in the ABTS assay—should be interpreted with caution. Because only one biological sample was available for each group, the calculated variances primarily reflect technical repeatability rather than biological variability, and part of the observed difference may fall within normal methodological fluctuations. Despite this limitation, the overall trend is consistent with the chemical profiling results (Figure 4), in which several flavonoids (e.g., vicenin-2, spinosin) and phenolic acids displayed increased relative abundances in ZSS-02. These constituents are widely recognized as major contributors to antioxidant activity in plant-derived oils. Previous studies have similarly reported that moderate thermal processing can promote the release of bound phenolics, facilitate glycosidic cleavage, or enhance the detectability of flavonoid aglycones, thereby improving the apparent antioxidant response [34]. In addition, heat-induced reactions, including mild oxidation and early Maillard processes, can generate amino-acid-derived intermediates and other Maillard reaction products with discernible radical-scavenging activity [35]. Taken together, the results suggest that stir-frying may induce compositional changes that modestly enhance the antioxidant response of ZSS oil. However, given that both ZSS samples exhibited IC_50_ values in the mg/mL range—substantially higher than that of ascorbic acid in the μg/mL range—the absolute antioxidant potency of ZSS oil remains limited. Therefore, the observed enhancement should be viewed as a relative improvement rather than a substantial increase in biological activity. Further investigations incorporating additional biological replicates and quantitative validation are necessary to confirm the robustness and physiological relevance of these findings.

In summary, the combined findings from non-volatile metabolite profiling, GC–IMS–based VOC characterization, and chemometric modeling collectively demonstrate that stir-frying induces coordinated thermal transformations in ZSS, resulting in detectable reorganization of both its volatile and non-volatile chemical components. These processing-related changes manifest not only as increased intensities of several aldehydes, ketones, and amino-acid–derived aroma compounds, but also as enhanced detectability of certain flavonoids and phenolic acids, reflecting the multiple thermal pathways—such as glycosidic cleavage, lipid degradation, and Maillard-type reactions—that act simultaneously during heating. Rather than indicating a uniform directional enhancement, these results highlight the complexity of heat-mediated reactions in lipid-rich botanical matrices and illustrate how different classes of constituents are differentially affected by stir-frying. From a traditional perspective, stir-frying is believed to achieve the goal of “nourishing nature and enhancing efficacy” through thermal modulation and aroma-mediated nutriregulation. By integrating modern analytical approaches, our study elucidates the scientific basis underlying these traditional practices and provides theoretical support and methodological guidance for the standardization of processing methods and the development of plant-derived functional oils.

## 5. Conclusions

This study integrated UPLC–Q–TOF–MS and GC–IMS, together with chemometric analyses, to characterize the compositional and aromatic changes that occur during the stir-frying of ZSS. The findings demonstrate that stir-frying induces measurable shifts in both non-volatile metabolites and volatile organic compounds, driven by processes such as glycosidic cleavage, lipid oxidation, and Maillard-type reactions. These transformations contribute to the enrichment of thermally derived aroma compounds and the increased detectability of certain flavonoids and phenolic acids. By combining GC–IMS with multivariate chemometric modeling, this study highlights the strong discriminatory power of VOC profiling for distinguishing processing conditions and identifying marker compounds associated with thermal transformation. These analytical strategies provide a valuable framework for future efforts aimed at evaluating processing consistency, supporting quality control, and refining the understanding of flavor-formation pathways in ZSS.

Several limitations should be acknowledged. The limited sample size and single-batch design restrict the generalizability of the findings, and the mechanistic relationships between specific metabolites and antioxidant responses warrant further verification using targeted assays. Future studies incorporating expanded sample sets, multi-omics platforms, and sensory evaluation will be essential for establishing comprehensive models of how processing modulates ZSS composition and function. Such work will help advance scientific, standardized processing protocols and contribute to the rational development of plant-derived functional oils.

## Figures and Tables

**Table 1:** Identification of chemical constituents of ZSS and stir-fried ZSS via UPLC-Q-TOF-MS.

No.	t/min	Compound Identification	Molecular Formula	Ion Form	Sample Presence	Measured *m*/*z*	Characteristic Fragment Ions (*m*/*z*)
1	0.902	Maltotetraose	C_24_H_42_O_21_	[M+FA-H]-	S>R	711	711; 665; 503; 341; 179
2	0.92	Gluconic acid	C_6_H_12_O_7_	[M-H]-	R>S	195	195; 129; 75
3	0.938	D-(+)-Raffinose ^(^^1)^	C_18_H_32_O_16_	[M+FA-H]-	S>R	549	549; 503 341; 221; 179
4	1.036	Trigonelline ^(1)^	C_7_H_7_NO_2_	[M+H]+	R>S	138	138; 92; 78; 65
5	1.201	Citric acid ^(1)^	C_6_H_8_O_7_	[M-H]-	S>R	192	191; 111; 87
6	1.328	L-Pyroglutamic Acid ^(1)^	C_5_H_7_NO_3_	[M-H]-	S>R	128	128; 85
7	1.454	Uridine ^(1)^	C_9_H_12_N_2_O_6_	[M-H]-	R>S	243	243; 200; 152; 110; 82
8	1.498	Nicotinamide ^(1)^	C_6_H_6_N_2_O	[M+H]+	S>R	123	123; 80; 53
9	1.641	1,4-Butanediyl bis[α-D-mannopyranoside]	C_16_H_30_O_12_	[M+FA-H]-	S>R	459	459; 413; 251; 179; 161
10	1.652	cAMP ^(1)^	C_10_H_12_N_5_O_6_P	[M+H]+	S-only	330	330; 136
11	1.759	Phenylalanine ^(1)^	C_9_H_11_NO_2_	[M+H]+	R>S	166	166; 120; 103; 91
12	2.043	Guanosine ^(1)^	C_10_H_13_N_5_O_5_	[M-H]-	S>R	282	282; 150; 133
13	2.232	Adenosine ^(1)^	C_10_H_13_N_5_O_4_	[M+H]+	S-only	268	268; 136; 119; 94
14	2.408	Verpacamide A	C_11_H_19_N_5_O_2_	[M+H]+	S>R	254	254; 195; 167; 125; 98; 70
15	2.618	Xanthosine ^(1)^	C_10_H_12_N_4_O_6_	[M-H]-	R>S	283	283; 151; 108; 80
16	3.111	L-Tryptophan ^(1)^	C_11_H_12_N_2_O_2_	[M-H]-	R>S	203	203; 116
17	3.521	/	C_14_H_21_N_5_O_3_	[M+H]+	S-only	308	308; 195; 167; 125; 98; 70
18	3.704	1-β-D-glucopyranosyl-2,3-dihydro-2-oxo-1H-Indole-3-acetic Acid ^(1)^	C_16_H_19_NO_8_	[M-H]-	S>R	352	352; 308; 190; 188; 146
19	4.413	/	C_46_H_52_N_2_O_4_	[M+NH4]+	S-only	714	714; 498; 313; 200; 158
20	4.589	Vanillic acid 4-O-β-D-glucoside	C_14_H_18_O_9_	[M-H]-	R>S	329	329; 167; 152; 123; 108
21	4.591	2-((R)-2-oxo-1-((2R,3R,4S,5S,6R)-3,4,5-trihydroxy-6-(hydroxymethyl)tetrahydro- 2H-pyran-2-yl)indolin-3-yl)acetic acid	C_16_H_19_NO_8_	[M-H]-	S>R	352	352; 308; 188; 160; 146
22	4.795	2,6-Dihydroxybenzoic acid 2-O-β-D-apiofuranosyl(1→2)-β-D- glucopyranoside	C_18_H_24_O_13_	[M-H]-	R>S	447	447; 315; 271; 152; 108
23	5.908	Dihydrophaseic acid 4′-O-β-glucopyranoside ^(1)^	C_21_H_32_O_10_	[M-H]-	R>S	443	443; 237; 189; 119
24	6.132	2,6-Dihydroxybenzoic acid 2-O-β-D-apiofuranosyl(1→2)-β-D- xylopyranoside	C_17_H_22_O_12_	[M-H]-	R>S	417	417; 285; 241; 152; 108
25	6.136	Coclaurine-6-glucoside	C_23_H_29_NO_8_	[M+H]+	R>S	448	448; 286; 269; 237; 175
26	6.321	/	C_44_H_43_N_3_O_2_	[M-H]-	S-only	644	644; 600; 470; 297; 279; 209; 128
27	6.749	Catechin ^(1)^	C_15_H_14_O_6_	[M-H]-	R>S	289	289; 245; 203; 123; 109
28	7.041	4-[(1,2,3,4-Tetrahydro-6,7-dimethoxy-2-methyl- 1-isoquinolinyl)methyl] phenyl β-D-glucopyranoside	C_25_H_33_NO_8_	[M+H]+	S-only	476	476; 314; 271; 269
29	7.65	Magnocurarine	C_19_H_24_NO_3_^+^	M+	S>R	314	314; 269; 175
30	7.735	N-Acetylphenylalanine	C_11_H_13_NO_3_	[M-H]-	S>R	206	206; 164; 147.; 103; 91; 72
31	7.74	(R)-6,8-di-β-D-glucopyranosyl-2,3-dihydro- 5,7-dihydroxy-2-(4-hydroxyphenyl)- 4H-1-Benzopyran-4-one	C_27_H_32_O_15_	[M-H]-	R>S	595	595; 475; 415; 385; 355
32	8.007	Coclaurine ^(1)^	C_17_H_19_NO_3_	[M+H]+	R>S	286	286.1445; 269.1186; 237.0917; 209.0963; 107.0498
33	8.122	(-)-Epicatechin ^(1)^	C_15_H_14_O_6_	[M-H]-	S>R	289	289.0733; 203.0742
34	8.786	Tembetarine	C_20_H_26_NO_4_^+^	M+	S>R	344	344; 301; 299; 267; 235; 175
35	9.551	Vicenin-2 ^(1)^	C_27_H_30_O_15_	[M-H]-	R>S	593	593; 503; 473; 383; 353
36	9.761	Magnoflorine ^(1)^	C_20_H_24_NO_4_^+^	M+	R>S	342	342; 297; 282; 265; 237
37	10.008	Lotusine	C_19_H_24_NO_3_^+^	M+	R>S	314	314; 269; 175
38	10.487	Apigenin 6,8-C-di-β-galactopyranoside	C_27_H_30_O_15_	[M-H]-	S-only	593	593; 503; 473; 413; 383
39	11.488	Isovitexin-2″-O-glucopyranoside	C_27_H_30_O_15_	[M-H]-	R>S	593	593; 413; 341; 293
40	11.872	Isospinosin	C_28_H_32_O_15_	[M-H]-	R>S	607	607; 487; 427
41	12.084	Spinosin ^(1)^	C_28_H_32_O_15_	[M-H]-	R>S	607	607; 427; 307
42	12.22	Azelaic acid ^(1)^	C_9_H_16_O_4_	[M-H]-	R>S	187	187; 169; 125; 123; 97
43	12.416	Zivulgarin	C_28_H_32_O_15_	[M-H]-	S-only	607	607; 427; 307
44	12.44	Isovitexin	C_21_H_20_O_10_	[M-H]-	R>S	431	431; 341; 311; 283
45	12.888	Apigenin-6-C-glucoside-7-O-glucoside	C_27_H_30_O_15_	[M-H]-	S>R	593	593; 413; 283; 269
46	12.9	Swertisin ^(1)^	C_22_H_22_O_10_	[M-H]-	R>S	445	445; 325; 297; 282
47	13.253	Fuzitine	C_20_H_24_NO_4_^+^	M+	S-only	342	342; 297; 282; 265; 237; 222; 191
48	13.585	Kaemperol-3-O-rutinoside ^(1)^	C_27_H_30_O_15_	[M-H]-	R>S	593	593; 285; 255
49	13.818	6″′-Vanilloylspinosin	C_36_H_38_O_18_	[M-H]-	S>R	757	757; 427; 209
50	14.242	6″′-p-Hydroxylbenzoylspinosin	C_35_H_36_O_17_	[M-H]-	S>R	727	727; 427; 179; 137
51	14.42	5,7-Dihydroxy-6-[2-O-[6-O-[(2E)-3-(4-hydroxy-3-methoxyphenyl)- 1-oxo-2-propen-1-yl]-β-D-glucopyranosyl]- β-D-glucopyranosyl]-2-(4-hydroxyphenyl)-4H-1-benzopyran-4-one	C_37_H_38_O_18_	[M-H]-	R>S	769	769; 593; 413; 293; 235
52	15.358	6″′-Sinapoylspinosin	C_39_H_42_O_19_	[M-H]-	R>S	813	813; 693; 607; 427; 325
53	15.669	6″′-Feruloylspinosin	C_38_H_40_O_18_	[M-H]-	R>S	783	783; 607; 427; 235
54	15.717	6″′-p-Coumaroylspinosin	C_37_H_38_O_17_	[M-H]-	R>S	753	753; 607; 427
55	15.756	6-[2-O-β-D-Glucopyranosyl-6-O-[2-[(3R)-1-β-D-glucopyranosyl- 2,3-dihydro-2-oxo-1H-indol-3-yl]acetyl]-β-D-glucopyranosyl]- 5-hydroxy-2-(4-hydroxyphenyl)-7-methoxy-4H-1-benzopyran-4-one	C_44_H_49_NO_22_	[M-H]-	R>S	942	942; 762; 589; 293
56	16.175	6-[2-O-β-D-Glucopyranosyl-6-O-[2-[(3S)-1-β-D-glucopyranosyl- 2,3-dihydro-2-oxo-1H-indol-3-yl]acetyl]-β-D-glucopyranosyl]- 5-hydroxy-2-(4-hydroxyphenyl)-7-methoxy-4H-1-benzopyran-4-one	C_44_H_49_NO_22_	[M-H]-	R>S	942	942; 762; 607; 589; 427; 352
57	16.669	5-hydroxy-8-[2-O-[6-O-[(2E)-3-(4-hydroxy-3-methoxyphenyl)-1-oxo- 2-propen-1-yl]-β-D-glucopyranosyl]-β-D-glucopyranosyl]- 2-(4-hydroxyphenyl)-7-methoxy-4H-1-benzopyran-4-one	C_38_H_40_O_18_	[M-H]-	S-only	783	783; 427; 235
58	16.846	5-Hydroxy-2-(4-hydroxyphenyl)-6-[2-O-[6-O-[3-(4-hydroxyphenyl)- 1-oxo-2-propen-1-yl]-β-D-glucopyranosyl]-β-D-glucopyranosyl]- 7-methoxy-4H-1-benzopyran-4-one	C_37_H_38_O_17_	[M-H]-	S-only	753	753; 607; 427
59	17.656	6″′-(-)-Phaseolspinosin	C_43_H_50_O_19_	[M-H]-	R>S	869	869; 839; 607; 427
60	18.261	6’’-Feruloyspinosin ^(1)^	C_38_H_40_O_18_	[M-H]-	R>S	783	783; 607; 427; 235
61	18.668	6-[6-O-[2-[(3S)-1-β-D-Glucopyranosyl-2,3-dihydro-2-oxo-1H-indol-3-yl]acetyl]- 2-O-[6-O-[3-(4-hydroxy-3-methoxyphenyl)-1-oxo-2-propen-1-yl]-β-D-glucopyranosyl]- β-D-glucopyranosyl]-5-hydroxy-2-(4-hydroxyphenyl)-7-methoxy-4H-1-benzopyran-4-one	C_54_H_57_NO_25_	[M+H]+	R>S	1120	1120; 782; 764; 665; 393; 327; 177
62	18.995	6-[6-O-[2-[(3R)-1-β-D-Glucopyranosyl-2,3-dihydro-2-oxo-1H-indol-3-yl]acetyl]- 2-O-[6-O-[3-(4-hydroxy-3-methoxyphenyl)-1-oxo-2-propen-1-yl]-β-D-glucopyranosyl]- β-D-glucopyranosyl]-5-hydroxy-2-(4-hydroxyphenyl)-7-methoxy-4H-1-benzopyran-4-one	C_54_H_57_NO_25_	[M+H]+	R>S	1120	1120; 782; 764; 665; 393; 327; 177
63	20.925	Amphibine D	C_36_H_49_N_5_O_5_	[M+H]+	R>S	632	632; 289; 261; 148
64	21.123	Frangufoline	C_36_H_42_N_2_O_2_	[M+H]+	R>S	535	535; 148; 133
65	23.013	Jujuboside A ^(1)^	C_58_H_94_O_26_	[M+Cl]-	R>S	1242	1241; 1205; 1073; 749
66	23.13	Pinellic acid	C_18_H_34_O_5_	[M-H]-	R>S	329	329; 229; 211; 171
67	23.422	Jujuboside A1	C_58_H_94_O_26_	[M+Cl]-	S>R	1242	1241; 1206; 1074; 749
68	23.761	Jujuboside B ^(1)^	C_52_H_84_O_21_	[M+FA-H]-	R>S	1090	1090; 1044; 912; 749
69	23.912	9,10,11-Trihydroxy-12-octadecenoic acid	C_18_H_34_O_5_	[M-H]-	R>S	329	329; 211; 171
70	24.034	9,12,13-Trihydroxy-10-octadecenoic acid	C1_8_H_34_O_5_	[M-H]-	R>S	329	329; 211; 171
71	24.045	Jujuboside B1	C_52_H_84_O_21_	[M+FA-H]-	R>S	1090	1090; 1044; 912; 749
72	24.099	(3β,16β,23R)-16,23:16,30-Diepoxy-20-hydroxydammar-24-en-3-yl O-6-deoxy-α-L-mannopyranosyl-(1→2)- O-[O-β-D-xylopyranosyl-(1→2)-6-O-acetyl-β-D-glucopyranosyl-(1→3)]- α-L-arabinopyranoside	C_54_H_86_O_22_	[M+FA-H]-	R-only	1131	1132; 1086; 1026; 954; 912; 893; 749
73	24.331	/	C_38_H_63_NO_10_	[M+Cl]-	S-only	728	728; 692; 522; 283; 265; 199
74	24.491	/	C_54_H_86_O_22_	[M+FA-H]-	S-only	1132	1132; 1086; 1026; 954; 893; 749
75	24.57	Dehydrophytosphingosine	C_18_H_37_NO_3_	[M+H]+	R>S	316	316; 298; 280
76	25.571	1-[(2S)-3-hydroxy-2-[[(9Z,12Z)-1-oxo-9,12-octadecadienyl]oxy]propyl hydrogen phosphate]-D-myo-Inositol	C_27_H_49_O_12_P	[M-H]-	R>S	595	595; 315; 279; 241; 153
77	25.742	D-myo-Inositol, 1-[(2R)-2-hydroxy-3-[(1-oxohexadecyl)oxy]propyl hydrogen phosphate]	C_25_H_49_O_12_P	[M-H]-	S>R	571	571; 315; 255; 241; 153; 79
78	26.017	1-[2-Hydroxy-3-[[(9Z)-1-oxo-9-octadecen-1-yl]oxy]propyl hydrogen phosphate]-myo-inositol	C_27_H_51_O_12_P	[M-H]-	R>S	597	597; 315; 281; 241; 153
79	26.094	1-Linoleoyl-sn-glycero-3-phosphorylcholine	C2_6_H_50_NO_7_P	[M+H]+	R>S	520	520; 502; 184; 104
80	26.675	1-Oleoyl-sn-glycero-3-phosphocholine	C_26_H_52_NO_7_P	[M+FA-H]-	R>S	566	566; 506; 281; 224
81	26.915	Epiceanothic acid	C_30_H_46_O_5_	[M-H]-	R>S	485	485; 439; 423
82	27.23	Cyclic pentaleucine	C_30_H_55_N_5_O_5_	[M+H]+	R>S	566	566; 340; 295; 227; 199
83	27.461	1-Oleoyl-sn-glycerol 3-phosphate	C_21_H_41_O_7_P	[M-H]-	S>R	435	435; 281; 152; 78
84	27.491	Gamma-Linolenoyl carnitine	C_25_H_43_NO_4_	[M+H]+	R>S	422	422; 376; 160; 114
85	27.992	O-Palmitoleoylcarnitine	C_23_H_43_NO_4_	[M+H]+	R>S	398	398; 352; 160; 114
86	28.009	Ceanothic acid	C_30_H_46_O_5_	[M-H]-	R>S	485	485; 423
87	28.403	O-linoleyl-L-carnitine	C_25_H_45_NO_4_	[M+H]+	R>S	424	424; 378; 160; 114
88	29.59	3β-O-(trans-p-Coumaroyl)maslinic acid	C_39_H_54_O_6_	[M-H]-	R>S	617	617; 453; 145
89	29.721	Betulinic acid ^(1)^	C_30_H_48_O_3_	[M+H-H2O]+	R>S	439	439; 393; 259; 241; 137
90	30.258	Oleamide	C_18_H_35_NO	[M+H]+	S>R	282	282.; 69
91	30.588	Oleic Acid	C_18_H_34_O_2_	[M+H]+	S-only	283	283; 135; 121; 107; 93
92	31.292	Soyacerebroside I	C_40_H_75_NO_9_	[M+H]+	R>S	714	715; 697; 534; 516; 262
93	32.997	D-myo-Inositol, 1-[(2R)-2-[(1-oxohexadecyl)oxy]- 3-[[(9Z,12Z)-1-oxo-9,12-octadecadienyl]oxy]propyl hydrogen phosphate]	C_43_H_79_O_13_P	[M-H]-	S>R	834	834; 553; 391; 279; 255; 241

Note: ^(1)^ verified by standards; “/”: unidentified; RP = raw only; SP = stir-fried only. Several metabolites appear in multiple ion forms (isomers, adducts, or fragment ions), which were consolidated and counted as single compounds. Entries marked as “/” denote features with valid molecular formulas and MS/MS fragments but lacking confident structural identification; such signals are reported as unidentified features. Sample presence codes: R-only: detected only in raw ZSS (ZSS-01); S-only: detected only in stir-fried ZSS (ZSS-02); R>S: present in both samples, higher in raw ZSS; S>R: present in both samples, higher in stir-fried ZSS.

**Table 2:** Analysis results for volatile oil components of ZSS.

NO	Compound	CAS	Formula	MW	RI	Rt/s	Dt/ms
1	γ-Butyrolactone	C96480	C_4_H_6_O_2_	86.1	1603	1184.01	1.08377
2	2-Acetyl-5-methylfuran	C1193799	C_7_H_8_O_2_	124.1	1601	1177.167	1.16054
3	Propanoic acid	C79094	C_3_H_6_O_2_	74.1	1561	1086.512	1.11613
4	methyl 2-furoate	C611132	C_6_H_6_O_3_	126.1	1558	1081.145	1.14754
5	Acetic acid-M	C64197	C_2_H_4_O_2_	60.1	1467	901.108	1.05442
6	Acetic acid-D	C64197	C_2_H_4_O_2_	60.1	1471	908.609	1.1589
7	2-Ethyl-5-methylpyrazine	C13360640	C_7_H_10_N_2_	122.2	1387	767.807	1.17362
8	1 -hexanol	C111273	C_6_H_14_O	102.2	1364	733.551	1.32865
9	2,5-Dimethylpyrazine	C123320	C_6_H_8_N_2_	108.1	1326	679.134	1.11963
10	(E)-2-Heptenal	C18829555	C_7_H_12_O	112.2	1331	684.874	1.25487
11	2-Butanone, 3-hydroxy-M	C513860	C_4_H_8_O_2_	88.1	1291	630.85	1.06623
12	2-Butanone, 3-hydroxy-D	C513860	C_4_H_8_O_2_	88.1	1292	633.923	1.3317
13	Cyclohexanone	C108941	C_6_H_10_O	98.1	1292	632.899	1.16192
14	1-octanal	C124130	C_8_H_16_O	128.2	1295	637.633	1.40796
15	2-Methylpyrazine	C109080	C_5_H_6_N_2_	94.1	1271	595.45	1.09174
16	1-Pentanol-M	C71410	C_5_H_12_O	88.1	1256	569.178	1.25342
17	1-Pentanol-D	C71410	C_5_H_12_O	88.1	1257	571.552	1.51276
18	3-Methylbutan-1-ol-M	C123513	C_5_H_12_O	88.1	1203	487.447	1.2419
19	3-Methylbutan-1-ol-D	C123513	C_5_H_12_O	88.1	1205	490.438	1.49425
20	(+)-limonene	C138863	C_10_H_16_	136.2	1192	472.182	1.21612
21	2-Heptanone	C110430	C_7_H_14_O	114.2	1179	454.127	1.26294
22	Heptaldehyde	C111717	C_7_H_14_O	114.2	1184	460.198	1.33668
23	2,5-Dimethyl thiophene	C638028	C_6_H_8_S	112.2	1175	447.978	1.0794
24	α-Terpinene	C99865	C_10_H_16_	136.2	1159	423.141	1.21508
25	Butanol-M	C71363	C_4_H_10_O	74.1	1139	396.254	1.18384
26	Butanol-D	C71363	C_4_H_10_O	74.1	1141	398.882	1.38147
27	3-Penten-2-one	C625332	C_5_H_8_O	84.1	1129	382.432	1.07735
28	Allyl sulfide	C592881	C_6_H_10_S	114.2	1126	379.317	1.11942
29	2-Pentanol-M	C6032297	C_5_H_12_O	88.1	1116	365.725	1.21353
30	2-Pentanol-D	C6032297	C_5_H_12_O	88.1	1117	367.424	1.44382
31	1-Butanol, 3-methyl-, acetate	C123922	C_7_H_14_O_2_	130.2	1121	372.521	1.30432
32	2-Methyl-1-propanol-M	C78831	C_4_H_10_O	74.1	1088	333.153	1.17268
33	2-Methyl-1-propanol-D	C78831	C_4_H_10_O	74.1	1089	333.742	1.36549
34	1-hexanal-M	C66251	C_6_H_12_O	100.2	1084	329.618	1.26829
35	1-hexanal-D	C66251	C_6_H_12_O	100.2	1085	330.502	1.5599
36	ethyl 3-methylbutanoate	C108645	C_7_H_14_O_2_	130.2	1068	314.592	1.26351
37	1-Propanol-M	C71238	C_3_H_8_O	60.1	1034	285.425	1.11053
38	1-Propanol-D	C71238	C_3_H_8_O	60.1	1034	285.719	1.24916
39	2-Methyl butanoic acid ethyl ester	C7452791	C_7_H_14_O_2_	130.2	1048	296.915	1.25076
40	2-butanol-M	C78922	C_4_H_10_O	74.1	1018	272.65	1.14903
41	2-butanol-D	C78922	C_4_H_10_O	74.1	1020	274.538	1.32737
42	n-Pentanal	C110623	C_5_H_10_O	86.1	981	245.833	1.18327
43	α-Pinene	C80568	C_10_H_16_	136.2	984	247.54	1.29202
44	2-Butanone	C78933	C_4_H_8_O	72.1	907	202.768	1.24549
45	Acetic acid ethyl ester	C141786	C_4_H_8_O_2_	88.1	891	194.507	1.33673
46	Butanal	C123728	C_4_H_8_O	72.1	888	192.666	1.11855
47	2-propanone	C67641	C_3_H_6_O	58.1	838	169.356	1.11489
48	Propanal-M	C123386	C_3_H_6_O	58.1	822	162.442	1.06094
49	Propanal-D	C123386	C_3_H_6_O	58.1	824	163.232	1.14507
50	Methyl acetate	C79209	C_3_H_6_O_2_	74.1	852	175.71	1.19289
51	2-Methyl propanal	C78842	C_4_H_8_O	72.1	836	168.291	1.2809
52	Ethyl 2-methylpropionate	C97621	C_6_H_12_O_2_	116.2	965	235.545	1.19807
53	2-Ethylfuran	C3208160	C_6_H_8_O	96.1	963	234.725	1.31265
54	γ-Terpinene	C99854	C_10_H_16_	136.2	1244	549.933	1.21443

Note: The substance suffixes M and D represent monomers and dimers of the same substance, respectively. MW refers to the molecular weight of each compound. Dt represents the drift time (ms) of the ion in the ion mobility spectrometry (IMS) dimension.

**Table 3:** Antioxidant activities of Ziziphi spinosae semen oils (ZSSOs) determined via DPPH and ABTS radical scavenging assays (n = 3).

Sample	DPPH IC_50_	ABTS IC_50_
ZSS-01	9.27 ± 0.84 ^a^ mg/mL	9.33 ± 0.25 ^a^ mg/mL
ZSS-02	5.74 ± 0.20 ^b^ mg/mL	8.12 ± 0.31 ^b^ mg/mL
Vc	6.83 ± 0.60 ^c^ μg/mL	2.80 ± 0.10 ^c^ μg/mL

Note: Values are expressed as means ± standard deviations (n = 3). Different letters within the same column indicate significant differences at *p* < 0.05.

## Data Availability

The original contributions presented in this study are included in this article; further inquiries can be directed to the corresponding authors.
